# Interpretation of DNA data within the context of UK forensic science — investigation

**DOI:** 10.1042/ETLS20210165

**Published:** 2021-06-21

**Authors:** Susan Pope, Roberto Puch-Solis

**Affiliations:** 1 Principal Forensic Services, Reading, U.K.; 2 Computer Science Department, Aston University, Birmingham, U.K.

**Keywords:** DNA data, forensic genealogy, forensic intelligence and prediction, forensic investigation

## Abstract

This article is the second part of a review of the interpretation of DNA data in forensic science. The first part describes the evaluation of autosomal profile for criminal trials where an evidential weight is assigned to the profile of a person of interest (POI) and a crime-scene profile. This part describes the state of the art and future advances in the interpretation of forensic DNA data for providing intelligence information during an investigation. Forensic DNA is crucial in the investigative phase of an undetected crime where a POI needs to be identified. A sample taken from a crime scene is profiled using a range of forensic DNA tests. This review covers investigation using autosomal profiles including searching national and international crime and reference DNA databases. Other investigative methodologies described are kinship analysis; familial searching; Y chromosome (Y-STR) and mitochondrial (mtDNA) profiles; appearance prediction and geographic ancestry; forensic genetic genealogy; and body identification. For completeness, the evaluation of Y-STRs, mtDNA and kinship analysis are briefly described. Taken together, parts I and II, cover the range of interpretation of DNA data in a forensic context.

## Introduction

DNA is a cornerstone of forensic science. Since its introduction into casework in the 1980s [1], it has benefited from a scientific basis in genetics for its production, and a probabilistic basis for its reporting. This document presents the state of the art and future for the interpretation of DNA data within the context of forensic cases. The content of this topic is vast and therefore the review has been divided into two parts.

This second part covers investigation, which also benefits from case assessment and interpretation (CAI) methodology which uses likelihood ratios (LRs) to rank investigative explanations. Investigation using autosomal STR profiles may identify a person of interest (POI) through searching national and international crime and reference DNA databases. Where the database searches are not sufficiently informative, the same data can be used to investigate kinship and familial relationships.

Avenues for an investigation that use other types of DNA data are also described. These provide other information about a person whose DNA is present in the crime stain. They include Y chromosome (Y-STR) and mitochondrial DNA (mtDNA) profiles; appearance prediction and geographic ancestry using single nucleotide polymorphisms (SNPs); and forensic genetic genealogy searching DNA databases produced from non-forensic DNA samples.

A different type of application for DNA intelligence is body identification which uses all these methodologies to identify a person whose remains have been recovered.

For completeness, the evaluation of Y-STRs, mtDNA and kinship analysis are briefly described.

## Investigation foundation

For evaluation, the DNA evidence consists of the reference profile of the POI and the crime-scene profile. The task is then to assign evidential weight to a pair of mutually exclusive propositions that are disputed at court. The propositions are organised in a hierarchy: sub-source, source, activity and offence. The propositions are anchored on the POI.

For investigation, in contrast, the DNA evidence consists only of the crime-scene profile. Investigation can also follow the CAI model [2,3]. Rather than propositions, explanations are put forward, which are organised using the same hierarchy. For each level of the hierarchy, a set of explanations ($H_{1},H_{2},\ldots ,H_{n}$) are proposed and ranked based on the crime-scene profile and the context of the investigation. The explanations are devised through abductive reasoning [4]. The forensic scientist uses her/his expertise for ranking of explanations according to their perceived likelihood and following the logical approach of Bayes’ theorem:

$$\begin{array}{rl} & \;\;\;\;\;\;\;\;\;\;\;\;\;\;\;\;\mathrm{Pr}(H_{i}|E,I)\propto \;P(E|H_{i},I)\;\times \;P(H_{i}|I)\\ & \text{Posterior probability }\propto \;\text{likelihood }\times \;\text{prior probability} \end{array}$$

That is, the posterior probability of the explanation ($H_{i})$ given the crime-scene profile (E) and the context of the case (I) is proportional to the probability of the crime-scene profile given $H_{i}$ and *I*, times the prior probability of the explanations given *I*. So, for evaluation, the likelihood ratio is the central quantity, but for investigation, the posterior odds for explanations is the central quantity.

Explanations are necessarily speculative, however, in order to make progress in an investigation, the risk of taking an action is more preferable to taking no action.

## Investigation using autosomal STR profiles

### Obtaining profile components for searching

A DNA profile is produced from a sample recovered from a crime scene and is displayed graphically as an electropherogram (epg) containing results at a set of regions of DNA called markers or loci. The DNA profile at a locus is recorded as a set of alleles, each of which is a small sequence of DNA called a short tandem repeat (STR) that is repeated many times, as described in the first part of this review [5].

There are two broad types of forensic investigation, one type uses DNA from human remains to identify the body, and the other uses DNA from a crime scene to provide intelligence information to identify a POI. For the first type, the aim is to obtain a profile from a single person. If the profile obtained is a mixture then further samples are taken until a single profile is obtained, although it may be incomplete. The resulting profile components are used in the search stage, described below.

**Figure 1.**
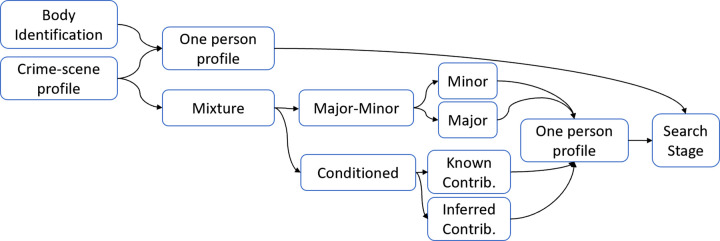
Investigation process for producing autosomal STR profiles to provide intelligence from crime-scene samples.

For the second type, the process starts when a DNA profile has been produced from a crime-scene sample, either from a body fluid or from sampling DNA in an area assumed to have been touched by the POI. The resulting crime-scene profile may be from one person, e.g. when a perpetrator breaks a window and cuts his hand, or it may be a mixture from several people, e.g. DNA obtained from a knife handle used by several people. Figure 1 shows a diagrammatic representation of the investigation process using autosomal STR profiles for both types.

If the profile so obtained comes from one person, the process can move to the search stage. However, if the profile is a mixture, there are some situations that may permit the inference of some of the components of the profile of one of the contributors.

One situation is where the mixture is unbalanced because one person has contributed most of the DNA. This is seen in the epg as a predominant contributor and it is referred to as the major profile. In this type of mixture, it may be possible to infer a second profile considering only the weaker or minor peaks. Both profiles can be used in the search stage although the minor profile may not be complete and may have originated from more than one person; therefore, the intelligence provided is less informative than the major profile search results. Another situation arises when the profile of a putative contributor is either known or can be inferred because of the history of the item. This is usually called the conditioning profile, e.g. the profile of the owner of the knife. An inferred conditioning profile may be based on a DNA profile from other items at the same scene even if they cannot be attributed to a known person. The conditioning profile enables several components of the remaining profile to be inferred, which can be used for the search stage.

**Figure 2.**
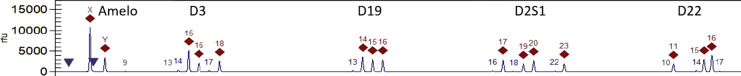
An epg of a balanced two-person mixture showing allele designations (red diamonds) and artefacts (blue triangles). The labels are the locus names.

For example, Figure 2 shows the sex test, Amelogenin (Amelo), and four loci of a balanced two-person mixture where both people contributed about the same amount of DNA. The loci in the figure are D3S1358 (D3), D19S433 (D19), D2S1338 (D2S1) and D22S1045 (D22). Let us assume that the conditioning profile is Amelo:X,X; D3:15,15; D19:15,16; D2S1:20,23; D22:11,16. At Amelo, a Y has been observed, so at least one male is present and therefore the unknown person is Amelo:X,Y. On the assumption that the profile is a two-person mixture, then the only option for the unknown person at D3 is 16,18. So, at this locus, the profile is fully determined. However, at D19 there are multiple options for the unknown person profile because we know only that it must include 14. This is usually written D19:14,F, where F is a wildcard representing any allele including another 14. Similarly, we can obtain D2S1:17,19 and D22:15,F. The entire inferred profile components are then used in the search stage.

A probabilistic genotyping system (PGS) [6] has the capability to infer a more informative set of genotype combinations. For example at D19, a PGS will assign probabilities to each of 14,14; 14,15 and 14,16. Only three possible genotype combinations are included, whereas D19:14,F represents many more combinations. The national DNA database (NDNAD) currently has the capability to run search queries D19:14,F and not a subset of possibilities. The subset of genotypes combinations can be used after the search to reduce the list of matches. PGS capability may be integrated into the search in the future.

### Search stage

Once the components of a one-person profile, the queried profile, have been determined, as described above, the intelligence information can be obtained by searching this against profiles from known people and from unsolved crimes held in the NDNAD [7]. If the queried profile is from an unidentified body, a search is carried out against the missing persons database, also held separately by the NDNAD. Note that the queried profile is the only information that is sent to the NDNAD.

If the queried profile meets the minimum standard for loading, it may be permanently loaded to the NDNAD. If it is below the loading standard, the search can still go ahead as a speculative or one-off search. These searches can be repeated at a later stage if no useful matches are obtained.

If a match is returned to a single known person, a report is sent to the investigating officer to review whether this maybe a POI in the case. When the queried profile is incomplete (usually called partial), with some loci containing the F wild card, multiple matches may be returned. Some of these will be adventitious (chance) matches. The forensic scientist may use the additional data in the epg, either visually or from a genotype combination list from a PGS, to exclude or rank the matches. For example in Figure 2, D19 was entered as 14,F but any return containing 14,14; 14,15 or 14,16; would be ranked higher than, for example, 14,20, because this is a good quality profile and no 20 allele has been observed. The ranked list is sent to the investigating officer who combines extra information from the case to determine whether any POI has been returned.

If a match is returned to a profile from an unsolved crime, it may provide intelligence information for the investigating officers of both cases because the information from one case may assist with the other case, and vice versa. The intelligence provided may be less informative when the information in both crime profiles is incomplete, depending on how much the designated alleles in the profiles overlap.

If no match is returned, international DNA databases may be searched. These are organised through Europol [8] and Interpol [9]. If matches are returned, these are handled in the same manner as with a search against the NDNAD. Another option may be to carry out a local mass screen of voluntarily provided samples. If no matches are returned by any of these methods for a serious case, i.e. murder or serial rapes, additional information may be extracted from the sample for further analysis. The current analytical options are Y-STR and mtDNA profiles, section ‘Y-STR and mtDNA profiles’. These provide further information for comparison once a POI has been identified.

The NDNAD will need to incorporate advances in DNA profiles. However, this would be a major change to the storage infrastructure and search algorithms.

### Kinship analysis

Another investigative approach is to use the DNA STR data to assist in identifying relatives of a body or the donor of a crime sample. The statistical methods underpinning this activity are well established [10,11]. Reference samples of putative relatives are taken, and profiles compared with that of the body or crime sample. An example of a pair of propositions addressed are

$H_{1}$: The DNA came from the son of Mr A and Ms B,

$H_{2}$: The DNA came from a person unrelated to Mr A and Ms B.

Note that the hypotheses are about the origin of the DNA, i.e. sub-source level [11], and not about how and when the DNA was deposited.

An LR is calculated using established kinship formulae [10,11] embedded into computer systems. The calculation uses allele presence only, in contrast with a PGS which also uses peak heights from the epg. An example of such a computer system is Familias [12–14]. It is also possible to use Bayesian Networks to perform these calculations [15,16].

Note that kinship analysis considers the relationship between small groups of profiles of proposed relatives, whereas familial searching and forensic genealogy are the interrogation of forensic and recreational DNA databases to suggest potential biological relatives.

In the future, kinship analysis will be supplemented by increased data from SNPs and massively parallel sequencing (MPS).

### Familial searching

Another search strategy is to conduct familial searching of forensic DNA databases. This is searching for potential biological relatives of the unknown donor of the crime-scene sample [17,18]. In practice, only two relationships are sufficiently informative when only using autosomal STRs employed in the NDNAD: parent/child and siblings. For parent/child, first a subset of profiles that shared at least one allele at each locus is selected. These are then ranked using case-specific information such as geography and age. For siblings, the entire database is ranked using LRs calculated from discrete statistical methods that only use allele absence/presence. The profiles are ranked further using case-specific information, usually addressed by the police, see [19,20] for a review. In the U.K., each case is reviewed by the Biometrics and Forensic Ethics Group before permission for a familial search is granted. This group reviews and balances the public safety and public good and the human rights of the people involved [21]. In the U.K., these searches are carried out infrequently: 17 searches in 2018/2019 and, 16 searches in 2019/2020 [7].

## Other investigative routes

### Y-STR and mtDNA profiles

A Y-STR profile is a haplotype produced from a set of loci on the male-specific Y chromosome, which is inherited through the paternal line. Typically, multiple loci are considered as a haplotype because they are inherited as a group. Current test systems include rapidly mutating loci which provides increased discrimination on closely related males [22]. Y-STR profiles have been used to screen male populations in relatively limited geographical areas. Such screens are informative about the paternal line, and not necessarily the identity, of the donor of the crime sample.

Y-STR profiles are usually used after a POI has been identified. The resulting Y-STR profile together with autosomal profiles gives a stronger evidential value. In future, Y-STRs may provide information about potential surnames of crime sample donors [23,24].

An mtDNA profile is produced from the mitochondria passed on by the mother to all offspring and are informative about the maternal line of a person. The interpretation and usability are similar to Y-STRs [25]. Y-STR and mtDNA profiles are used as secondary tests to support body identification.

### Predicting appearance and biogeographic ancestry

Methods for predicting physical appearance are being developed. These use predictive markers for appearance, ancestry as well as chronological age. The technology used is called forensic DNA phenotyping (FDP) which is similar to MPS. There are several platforms like VISAGE, SnapShot and Hirisplex. The VISAGE consortium is partners from academic, police and justice institutions of eight European countries funded by a European Union framework programme [26,27]. VISAGE aims to construct facial characteristics of unknown perpetrators based on the crime-scene sample. Parabon Nanolabs uses SnapShot for similar purposes [28,29]. Hirisplex is developed by [30] to predict eye, hair and skin colour [30,31]. The legal and ethical aspects of these methodologies are still being developed [32]. For a review of these methods, see [33]. FDP was used in a recent criminal case in Germany where the existing legislation was updated to allow its application [34].

## Forensic genealogy

From the early 1970s to the mid-1980s, a series of apparently unconnected rapes and murders remained unsolved in California. Crime-scene samples were re-tested in the 1990s using newly introduced forensic DNA techniques, which showed that many of these crimes were committed by the same perpetrator who became known as the Golden State killer [35]. However, no matches were returned by conventional searches (section ‘Search stage’) of forensic DNA databases.

In the meantime, people started investigating their ancestry as a hobby using DNA tests developed for non-forensic uses. These services are provided direct-to-consumer by commercial companies, e.g. Ancestry.com, 23andme and FTDNA, who maintain searchable databases of the customer test results. In 2018, the Golden State Killer was apprehended using a new methodology based on searching these recreational ancestry databases [36].

**Figure 3.**
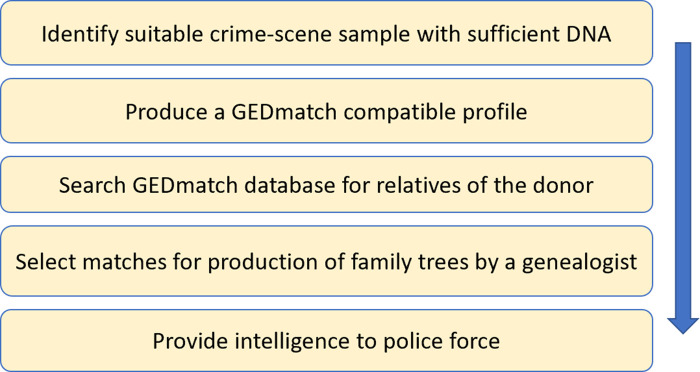
Stages of forensic genetic genealogy.

This methodology, subsequently called forensic genealogy, can be applied when there is sufficient unused DNA from a crime-scene sample after forensic testing has not yielded any matches. The stages of the methodology are shown in Figure 3. Ideally, the DNA is of good quality from one person.

The genealogy DNA test looks at variations of SNPs which are tested in large groups or arrays typically covering more than 600 thousand sites across the entire genome for all chromosomes and mitochondria. This raw DNA data can be loaded to open source databases such as GEDmatch Genesis (now part of Verogen Inc.) as a GEDCOM file. This enables different providers to load data of their customer into a single searchable database. A measure of the similarity between profiles, in centimorgans (cMs), is calculated between the crime-scene profile and each profile in the database. The larger the similarity measure, the closer the relationship of the donor.

**Figure 4.**
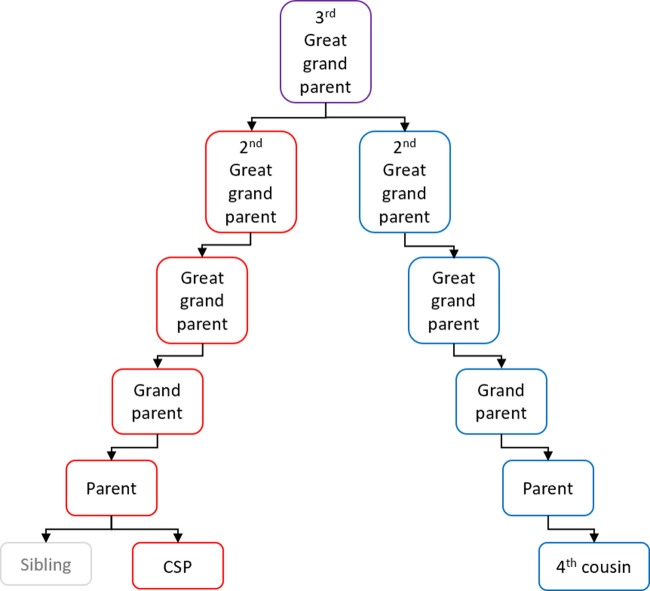
A family tree showing a fourth cousin relationship.

The profiles in the ancestry database are sorted according to the similarity measure in descending order of relatedness. A subset of profiles at the top of the list is selected for further analysis. The list is organised into groups of relatives who may descend from a common ancestor using information already contained in the database. The list is then passed to a genealogist whose role is to find or build family trees, e.g. Figure 4, for the clusters using publicly available information, e.g. existing public family tree sites, birth, marriage and death certificates, and census records. The aim of a tree is to indicate a common ancestor of the clusters and the crime-scene profile.

Once common ancestors are determined, the genealogist determines descendants that may be currently alive. The whole process is labour intensive and can take hundreds of hours.

The descendant list may identify a POI or a close relative of the POI who can be asked to provide a DNA sample. In the U.K. if there is sufficient information to arrest the POI, they can request a DNA sample from him. In the U.S.A. there is no legal requirement for the POI to provide a DNA sample and a surrogate profile is sought from, for example, cigarette ends or a drinking glass.

Forensic genealogy can be provided by commercial companies, e.g. Parabon Nanolabs who employ genealogists. There are few peer-reviewed publications on the accuracy of the identification. One example is [37]. It is possible, as with any investigative work, that the outcomes may be misleading [38] because of misinterpretation of artefacts. False positives where a relationship had been asserted incorrectly have been seen [39]. There was also a breach of the GEDCOM security by hackers [40]. There are discussions about the ethics of using uncontrolled data provided by customers without a legal framework and without any quality control [41,42]. Changes to the GEDmatch terms and conditions and a technical error in January 2021 allowed the Database to be searched by Police regardless of whether the user had expressly given permission for this [43]. However, guidelines for this are being developed [44,45].

Currently, in the U.K., no forensic genealogy searches are recorded in the NDNAD report [7]. The NDNAD Delivery Unit commissioned a review by the Biometrics and Forensics Ethics Group on the feasibility of such searches which concluded that the usefulness is limited because the ancestry databases are not U.K. based, and was concerned that the process is unregulated [46].

## Body identification

Body identification refers to the determination of the identity of human remains. DNA is one of the scientific methods used for this purpose, along with dental records and fingerprints. DNA is particularly useful when the remains are decomposed or consist of partial body parts. The identification is an essential part of the investigative process which may later lead to a criminal investigation. An autosomal profile obtained from the remains can be searched against available DNA databases, section ‘Search stage’. Unidentified profiles may be added to the missing persons database.

Familial searching and kinship analysis, section ‘Investigation Using Autosomal STR Profiles’, may be used when there is prior information (e.g. clothing, tattoos, passenger records, identification by relatives) suggesting a potential identity for the remains [47]. Forensic genealogy, section ‘Forensic Genealogy’, is also being used, e.g. the DNA Doe project [48] and The Centre for Human Identification [49] in the U.S.A.

The magnitude of the LR required for identification depends on the nature of the incident, e.g. a plane crash on a deserted area has a list of passengers, surrogate profiles and profiles from relatives. Therefore, prior information can be used to limit the number of possible identifications. In contrast, in an earthquake less prior information is available and thus, higher LRs are required. Kinship analysis was employed to identify the remains after the World Trade Center attack [50,51].

On a larger scale, the International Commission on Missing Persons is an intergovernmental organisation that addresses the investigation of bodies recovered as a result of armed conflicts, violations of human rights, and natural disasters [52], using the methods described above [53].

## Evaluation of Y-STR and mtDNA profiles

### Y-STR profiles

A Y-STR profile will typically be used in the investigation of sexual offences such as rape, section ‘Y-STR and mtDNA profiles’. For evaluation, the process is similar to the evaluation of profiles, however, there are some differences. Whereas for autosomal profiles the sub-source level propositions would be

$H_{1}$: The POI is the source of the DNA,

$H_{2}$: Someone unrelated to the POI is the source of the DNA,

for the evaluation of Y-STR profiles, they would be [54],

$H_{1}$: The POI or a male from their paternal lineage is the source of the DNA,

$H_{2}$: Neither the POI nor any male from their paternal lineage is the source of the DNA.

Another difference lies in the calculation of evidential weight. The POIs profile is compared against the Y-STR profile from a crime-scene sample. A match can be evaluated by comparing the POI's Y-STR profile against a collected dataset called YHRD [55,56]. Research on the statistical evaluation of Y-STR profiles has been active recently [57–60]. However, there is no single generally agreed method.

### Mitochondrial profiles

Another type of DNA profile is that obtained from the mitochondria of a cell. The DNA of the mitochondria (mtDNA) is separate from autosomal DNA and is inherited by sons and daughters from the maternal line. There are thousands of copies in each cell and it survives harsh treatment such as fire. For example, in a case involving skeletal remains or remains recovered after a fire, this DNA is more likely to be of use for profiling.

mtDNA can be used for body identification by obtaining a profile from the body and compare it against the mtDNA profile of putative relatives. It has some evidential value when a POI has been identified. The POIs profile is compared against the mtDNA profile from a crime-scene sample. A match can be evaluated by comparing the sequence against a collected dataset called EMPOP [61].

Although these tests are not very powerful, they provide some intelligence and evidential value in situations where autosomal DNA is insufficient.

In future, Y-STR and mtDNA tests will be incorporated in MPS arrays and they will become routine rather than specialist work.

## Evaluative kinship analysis

The calculations involved in kinship analysis for evaluation and investigation are the same for both purposes (section ‘Kinship analysis’). The difference is that the DNA is anchored on the POI instead of from the body or a crime scene. An example of the hypotheses of interest is:

$H_{1}$: The POI, the father of Ms B, and Ms B are the parents of the child,

$H_{2}$: An unknown male and Ms B are the parents of the child.

## Summary

- In forensic science, DNA data is interpreted to provide intelligence and assistance to the investigator determining the identity of the person that DNA in a crime stain originated from and how it was deposited.
- CAI gives a structure that clarifies the investigative requirements and mitigates against cognitive bias.
- Autosomal profiles from a single source and mixed profiles can be interpreted using PGS to derive results suitable for searching Forensic DNA Databases.
- Other investigative approaches are familial searching of Forensic DNA Databases, kinship analysis, body identification and Forensic Genetic Genealogy.

## Abbreviations

CAI: case assessment and interpretation
FDP: forensic DNA phenotyping
LRs: likelihood ratios
MPS: massively parallel sequencing
NDNAD: national DNA database
PGS: probabilistic genotyping system
POI: person of interest
SNPs: single nucleotide polymorphisms
